# Pupillometry in the follow-up of patients undergoing EVT - prediction of space-occupying hemispheric infarction

**DOI:** 10.1007/s00415-023-11797-w

**Published:** 2023-06-10

**Authors:** Clara-Sophie Kossel, Franca Kobus, Matthias C. Borutta, Maximilian Kärtner, Joji B. Kuramatsu, Tobias Engelhorn, Stefan Schwab, Julia Koehn

**Affiliations:** 1 Department of Neurology, Friedrich-Alexander-University of Erlangen-Nuremberg, Schwabachanlage 6, 91054 Erlangen, Germany; 2 Department of Neuroradiology, University of Erlangen-Nuremberg, Schwabachanlage 6, 91054 Erlangen, Germany

**Keywords:** Automated pupillometry, Pupillary reactivity, Ischemic stroke, Large vessel occlusion, Endovascular treatment

## Abstract

**Background:**

Despite benefits of endovascular treatment (EVT) for large vessel occlusion (LVO) ischemic stroke, space-occupying brain edema (BE) represents a detrimental complication. In critical-care settings, CT-imaging is needed for monitoring these patients. Yet, bed-side techniques with the potential to predict whether patients develop BE or not would facilitate a time- and cost-efficient patient care. We assessed clinical significance of automated pupillometry in the follow-up of patients undergoing EVT.

**Methods:**

From 10/2018 to 10/2021, neurocritical-care-unit patients were retrospectively enrolled after EVT of anterior circulation LVO. We monitored parameters of pupillary reactivity [light-reflex-latency (Lat), constriction- and redilation-velocities (CV, DV), percentage-change-of-apertures (per-change); NeurOptics-pupilometer^®^] up to every hour on day 1–3 of ICU stay. BE was defined as midline shift ≥ 5 mm on follow-up imaging 3–5 days after EVT. We calculated mean values of intra-individual differences between successive pairs of parameters (mean-deltas), determined best discriminative cut-off values for BE development (ROC-analyses), and evaluated prognostic performance of pupillometry for BE development (sensitivity/specificity/positive-/negative-predictive-values).

**Results:**

3241 pupillary assessments of 122 patients [67 women, 73 years (61.0–85.0)] were included. 13/122 patients developed BE. Patients with BE had significantly lower CVs, DVs, and smaller per-changes than patients without BE. On day 1 after EVT mean-deltas of CV, DV, and per-changes were significantly lower in patients with than without BE. Positive-predictive-values of calculated thresholds to discriminate both groups were considerably low, yet, we found high negative-predictive-values for CV, DV, per-changes, and mean-deltas (max.: 98.4%).

**Conclusion:**

Our data suggest associations between noninvasively detected changes in pupillary reactivity and BE early after LVO-EVT. Pupillometry may identify patients who are unlikely to develop BE and may not need repetitive follow-up-imaging or rescue-therapy.

## Introduction

Hemispheric infarctions due to large vessel occlusion (LVO) are associated with high rates of morbidity and mortality [1]. The development of severe space-occupying brain edema (BE) following the breakdown of membrane function and the blood–brain barrier is a major contributor to poor outcome following LVO of the anterior circulation [2]. BE and neurological deterioration usually occur during the first 24–48 h after symptom onset [3] and treatment options, e.g., decompressive hemicraniectomy, should be considered as early as possible to maximize chances of survival and favorable outcome [4].

While deteriorating levels of consciousness often represent first clinical signs of impending BE in not-sedated patients [3], early recognition in comatose, mechanically ventilated patients on neurological intensive-care units (ICU) is subject to additional diagnostic tools. Although alternative sedation protocols have been emphasized to be considered over general anesthesia (GA) during thrombectomy [5], and periprocedural conscious sedation concepts have been associated with improved functional outcome [5], there is still a notable number of LVO patients who receive GA in order to minimize procedural risk, e.g., patients with aphasia-associated limited comprehension. Furthermore, especially in more severely affected patients with initial impairment of consciousness, GA may still be necessary, and patients may stay on ventilator support for some time after endovascular treatment (EVT) [6, 7] particularly in those cases when complications like BE are expected due to long time-windows or absent recanalization success. Thus, several LVO patients may stay on ventilator support for some time after EVT [6], complicating clinical evaluation.

After thrombectomy, routine control imaging is usually not performed earlier than the next day. Meanwhile, the actual infarction size remains unknown to the treating physicians [8], However, patients with large hemispheric infarctions (LHI) and developing BE would benefit from early detection and subsequent treatment decisions [4]. After ICU admission, repeated neuroimaging is the gold standard for monitoring sedated patients [9]. However, transportation of critically ill patients to the radiology department is associated with an increased risk of complications [10], and cumulative radiation doses can reach considerably high levels [11]. For a safety-, time-, and cost-efficient patient care, simple bed-side techniques with the potential to reliably predict BE already within the early phase after EVT would be desirable.

An abnormal pupillary light reflex has been demonstrated to be a valid indicator for a rise in intracranial pressure (ICP) due to developing BE [12–14]. Regular assessments of pupillary function are therefore an inherent part of patient monitoring on neurological ICUs [14]. Manual pupillary assessments, however, have been shown to be subject to large inter-examiner variability [15] and vulnerable to external conditions, e.g., lighting [12]. Automated pupillometers, presumed to be indifferent to those external influences, are increasingly becoming part of standard monitoring on neurological ICUs and the prediction of a rise in ICP after traumatic brain injury, intracranial bleeding, or ischemic infarction using the automated system seems plausible [13, 14, 16–18].

Yet, there are only few studies assessing the prognostic performance of automated pupillometry in ischemic stroke patients, and the exact time-point of pupillary light reflex deterioration in relation to EVT has so far not been analyzed in these studies [13, 14, 19]. The clinical value of this non-invasive technique especially in the early phase after EVT in LVO patients is therefore still not fully evaluated.

The aim of this study was to investigate the prognostic value of automatically assessed dynamic pupillary parameters in those patients at specific time-points after EVT, and to establish potential prediction parameters for a reliable non-invasive and bed-side differentiation whether patients are at risk of developing space-occupying BE or not—even before control imaging revealed the actual infarction size.

## Methods

### Patient-selection

All patients with anterior circulation LVO admitted to the neurological ICU of the University Hospital Erlangen, Germany, between 10/2018 and 10/2021 were screened for eligibility to participate in this retrospective study. Patients met the inclusion criteria if they (i) underwent EVT, (ii) required sedation and mechanical ventilation on a certified ICU, and (iii) did not show signs of orbital trauma, structural eye abnormalities, or absent light reflex, when manually assessed. All available data on pupillary parameters during the first 3 days of ICU stay were analyzed if clearly assigned to a certain time-point. Algorithms for treatment of BE and/or midline shift (MLS) were left to the judgment of the treating physicians. The institutional review board approved innocuousness of the study protocol.

### Data-assessment

Data on demographic parameters (age, sex), prior medical history, clinical status on admission (National Institute of Health Stroke Scale score [NIHSS]), and treatment of BE and/or MLS were retrieved from the institutional electronic-databases. Diagnosis of LVO was made upon cranial computed tomography (CT) including CT-angiography (CTA) and CT-perfusion-imaging (CTP) (SOMATOM Definition AS + , Siemens Healthineers, Forchheim, Germany). CT-scans performed upon hospital admission as well as follow-up CT-scans 3–5 days after admission were included into the study. Neuroimaging data were retrospectively analyzed by an independent, specialized neuroradiologist for presence of MLS, defined as ≥ 5 mm deviation of the septum pellucidum from the ideal midline between the most anterior and posterior part of the falx cerebri, as corresponding CT-scan finding of significant BE.

Quantitative pupillometry data were obtained using NeurOptics^®^ pupillometers (NeurOptics, Irvine, CA, USA). Over a period of 3 s, the pupillometer automatically analyzes two static [pupil size and minimum aperture (mm)], and four dynamic [light-reflex latency (Lat) (s), constriction- (CV) and redilation-velocities (DV) (mm/s), and percentage-change-of-apertures (per-change) (%)] pupil variables [12]. According to the institutional protocol, the treating physicians or bed-side nurses monitored pupillary reactivity every 60 min during the first 24 h, and every 180 min during the following 48 h of the ICU stay. Parameters of the eye ipsilateral to LVO were used for analyses. For early detection of abnormalities in pupillary function as sign of imminent neurological deterioration, differences between successive pairs of the assessed pupillary parameters in each individual patient (e.g., the second minus the first measured value of the parameter CV) were calculated as intra-individual “delta”. To narrow down the time-point when pupillary abnormalities become apparent following LVO-EVT, we further calculated mean values of intra-individual deltas on day 1 of monitoring.

### Statistical analysis

A commercially available statistical program (IBM, SPSS Statistics 28) was used for data analysis. Significance was set at *p* < 0.05. Data are expressed as median with interquartile range, or mean ± standard deviation, according to distribution of variables. For analysis of baseline characteristics and pupillary parameters, patients were dichotomized according to the presence of MLS upon follow-up CT. We used the nonparametric Mann–Whitney *U* test for unpaired samples for not normally distributed variables. Frequency distributions of categorical variables (percentage) were compared by Yates-Chi^2^ and Fisher’s-exact-probability tests. We performed receiver-operating-characteristics (ROC) analyses to investigate associations of BE, with pupillary parameters, and to determine best discriminative cut-off values for prediction of developing BE. Finally, prognostic performance of calculated cut-off values was assessed by determining sensitivity, specificity, and positive- and negative-predictive-values (PPV, NPV). Corresponding Odds-Ratios (ORs) with 95% Confidence Intervals (CIs) were calculated using the efficient-score method (corrected for continuity) as described elsewhere [20].

## Results

### Baseline characteristics

**Fig. 1:**
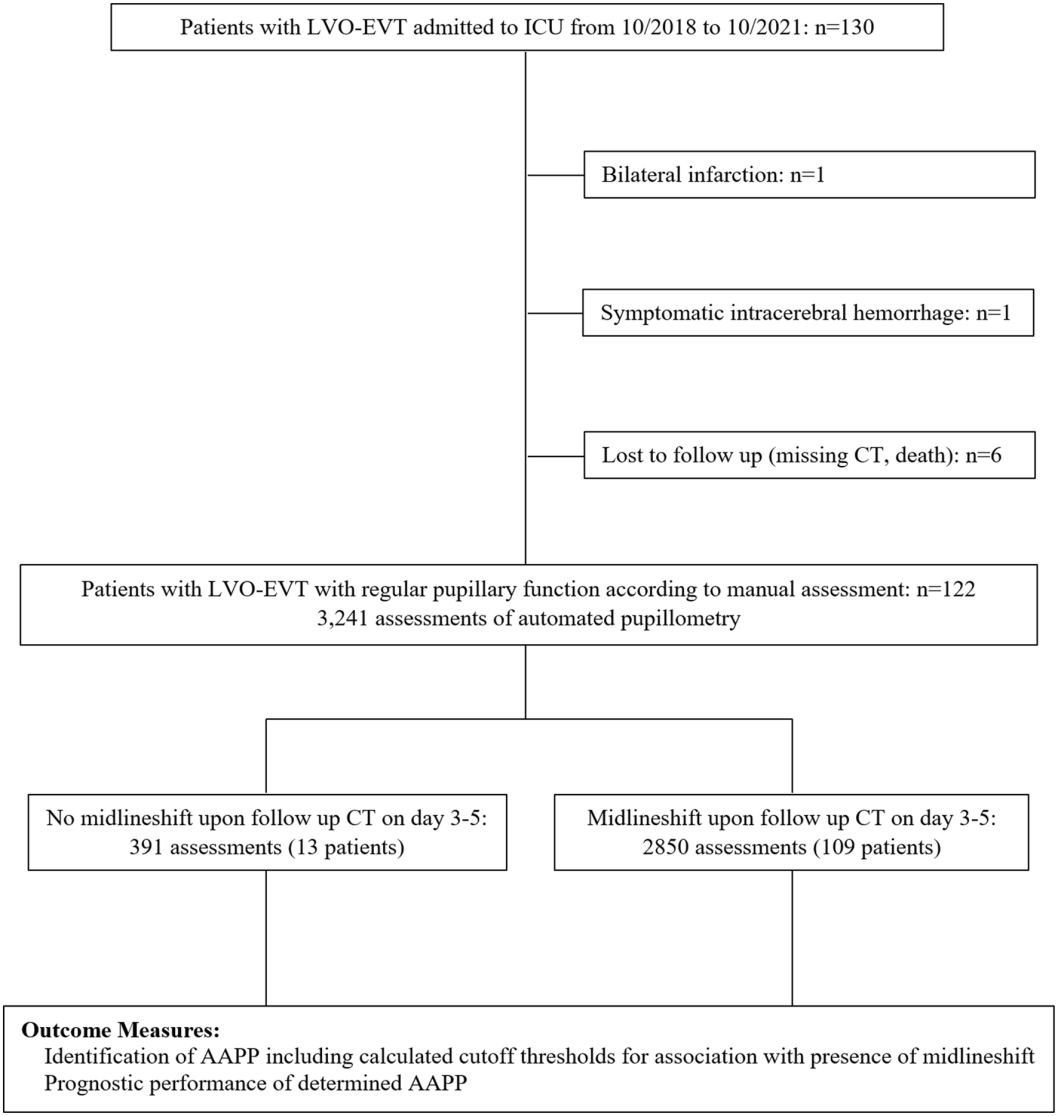
Flowchart of study participants. Overall, 130 patients with endovascular treatment (EVT) due to large vessel occlusion (LVO) of the anterior circulation admitted to the ICU between October 2018 and October 2021 were screened for eligibility. After exclusion of 1 patient because of bilateral infarction, 1 patient because of symptomatic intracerebral hemorrhage, and 6 patients that were lost to follow up (missing CT-scan, death), 122 patients and 3,241 assessments of automated pupillometry were available for data analysis. *AAPP*, automatically assessed pupillary parameters; *CT*, computed tomography; *EVT*, endovascular treatment; *LVO*, large vessel occlusion; *ICU*, intensive-care unit

Over a 3-year period, a total of 122 patients [67 women, median age 73.0 (61.0–85.0) years] with EVT due to LVO of the anterior circulation were enrolled (Fig. 1). Overall, there were 3,241 pupillary readings available [Fig. 1; median number of assessments per patient: 27 (16–38)]. Clinical baseline characteristics of patients with and without MLS are presented in Table 1. MLS on follow-up CT 3–5 days after admission was present in 13/122 patients (10.7%). There was no significant difference between the two patient groups regarding age, sex, admission NIHSS, number of pupillometry measurements, and side of LVO (Table 1). However, osmotic therapy (i.e., mannitol and/or hypertonic saline) was initiated significantly more often in patients with MLS, and the number of patients with a TICI (thrombolysis in cerebral infarction) score of 2a or less was significantly higher in our group with MLS compared to the group without MLS (30.8% versus 6.4%, *p* = 0.0155, Table 1).

**Table 1:** Baseline and clinical characteristics of 122 patients with endovascular treatment (EVT) due to large vessel occlusion (LVO) of the anterior circulation, according to presence of midline shift upon follow-up CT on day 3–5 post-stroke

	Midline shift ≥ 5 mm upon follow-up CT on day 3–5		
	No	Yes	*p* value
Patients, *n* (%)	109 (89.3)	13 (10.6)	
Age (mean ± SD; years)	74 (± 12)	69 (± 15)	0.293
Female gender, *n* (%)	61 (56)	6 (46.2)	0.708
NIHSS on admission (mean ± SD)	16 (± 8)	16 (± 5)	0.392
Number of pupillometry assessments (mean ± SD)	26 (± 11)	30 (± 10)	0.301
Side of LVO = left hemisphere, *n* (%)	57 (52.3)	9 (69.2)	0.386
Osmotic therapy, *n* (%)	12 (11.0)	9 (69.2)	** < 0.001**
Number of patients with TICI score < 2b, *n* (%)	7 (6.4)	4 (30.8)	**0.0155**

*EVT*, endovascular treatment; *LVO*, large vessel occlusion; *TICI*, thrombolysis in cerebral infarction; *n*, number; *SD*, standard deviation. Bold indicates signifcant diferences between patients with and without MLS

### Association between pupillary parameters and midline shift upon follow-up CT

**Fig. 2:**
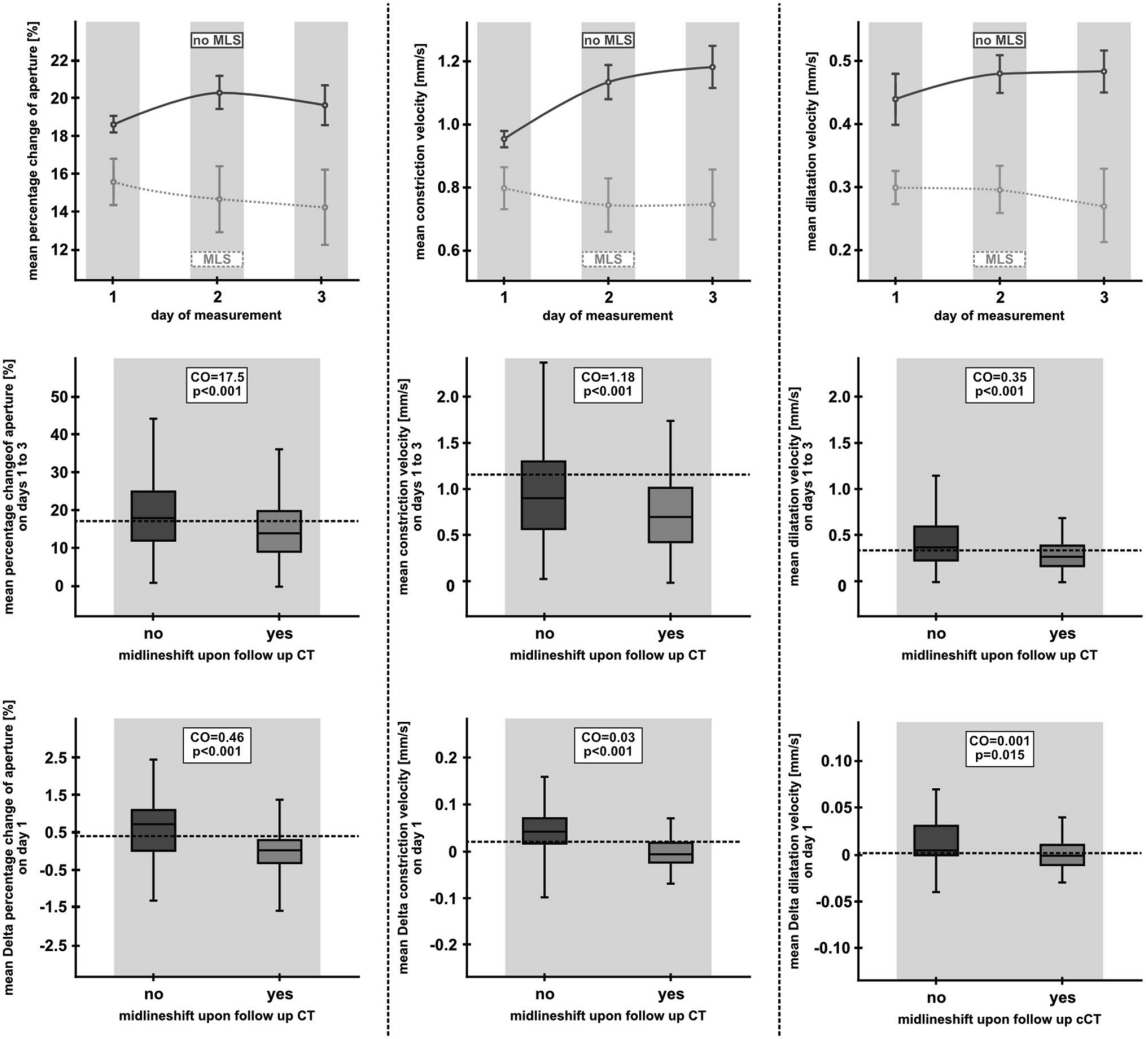
Automated pupillometry readings in relation to the presence of midline shift upon follow-up CT on day 3–5 after endovascular treatment (EVT) due to large vessel occlusion (LVO) ischemic stroke in 109 patients without (black) and 13 patients with (gray) midline shift. Percentage-change-of-aperture (upper left graph), constriction velocity (upper middle graph), and dilation velocity (upper right graph) on days 1, 2, and 3 after LVO-EVT. Mean values of percentage change of aperture (mid-left graph), constriction velocity (mid middle graph), and dilation velocity (mid-right graph) on days 1–3 after LVO-EVT. Mean values of intra-individual differences between successive pairs of automatically provided pupillometry parameters on day 1, Mean-Delta percentage change of aperture (lower left graph), Mean-Delta constriction velocity (lower middle graph), and Mean-Delta dilation velocity (lower right graph). *CO*, calculated best discriminative cutoff; *CT*, computed tomography

On day 1 after EVT, day 2, day 3, and during the entire monitoring period, i.e., days 1–3, all recorded parameters but Lat were significantly smaller in patients with than without MLS (Table 2, Fig. 2). Moreover, mean-*Deltas* of all parameters except Lat were significantly smaller in patients with than without MLS on the first day of monitoring (Table 3, Fig. 2).

**Table 2:** Group comparison of automatically provided dynamic pupillometry parameters in 122 patients with endovascular treatment (EVT) due to large vessel occlusion (LVO) of the anterior circulation, according to the presence of midline shift upon follow-up CT on day 3–5 post-stroke

	Midline shift ≥ 5 mm upon follow-up CT on day 3–5		
	No	Yes	*p* value
Day 1 after EVT of anterior circulation LVO			
Per-change [%] (IQR)	18.61 (12.0–24.0)	15.57 (9.0–21.0)	** < 0.001**
CV [mm/s] (IQR)	0.95 (0.55–1.22)	0.80 (0.42–1.01)	** < 0.001**
DV [mm/s] (IQR)	0.44 (0.22–0.56)	0.30 (0.17–0.41)	** < 0.001**
Lat [s] (IQR)	0.26 (0.23–0.27)	0.25 (0.23–0.27)	0.5
Mean-*Delta* per-change [%] (IQR)	0.78 (0.20–1.13)	0.02 (-0.32–0.42)	**0.003**
Mean-*Delta* CV [mm/s] (IQR)	0.05 (0.02–0.07)	-0.01 (-0.02–0.02)	** < 0.001**
Mean-*Delta* DV [mm/s] (IQR)	0.03 (0.0–0.04)	0.01 (-0.01–0.02)	**0.014**
Mean-*Delta* Lat [s] (IQR)	0.0 (-0.01–0.0)	0.0 (-0.01–0.0)	0.586
Day 2 after EVT of anterior circulation LVO			
Per-change [%] (IQR)	20.28 (13.5–27.0)	14.65 (9.75–20.0)	** < 0.001**
CV [mm/s] (IQR)	1.13 (0.72–1.45)	0.74 (0.48–1.02)	** < 0.001**
DV [mm/s] (IQR)	0.48 (0.25–0.65)	0.3 (0.19–0.39)	** < 0.001**
Lat [s] (IQR)	0.25 (0.23–0.27)	0.24 (0.20–0.27)	0.342
Day 3 after EVT of anterior circulation LVO			
Per-change [%] (IQR)	19.61 (13.0–25.0)	14.22 (10.0–18.0)	** < 0.001**
CV [mm/s] (IQR)	1.18 (0.73–1.46)	0.75 (0.46–1.05)	** < 0.001**
DV [mm/s] (IQR)	0.48 (0.26–0.64)	0.27 (0.16–0.35)	** < 0.001**
Lat [s] (IQR)	0.26 (0.23–0.30)	0.23 (0.2–0.30)	0.199
Days 1–3 after EVT of anterior circulation LVO			
Per-change [%] (IQR)	19.03 (12.0–25.0)	15.14 (9.0–20.0)	** < 0.001**
CV [mm/s] (IQR)	1.01 (0.58–1.31)	0.78 (0.45–1.02)	** < 0.001**
DV [mm/s] (IQR)	0.45 (0.23–0.60)	0.29 (0.17–0.38)	** < 0.001**
Lat [s] (IQR)	0.26 (0.23–0.27)	0.25 (0.23–0.27)	0.119

*EVT*, endovascular treatment; *LVO*, large vessel occlusion; *IQR*, interquartile range; *mm*, millimeter; *s*, second; *mean delta*, intra-individual differences between successive pairs of assessed pupillary parameters; *Per-change*, percentage change of aperture, i.e., maximum size minus minimum size divided by maximum size; *CV*, constriction velocity, i.e. amount of constriction divided by the duration of the constriction; *DV*, dilation velocity, i.e. amount of pupil size recovery divided by the duration of the recovery; *Lat*, light reflex latency, i.e. time difference between initiation of retinal light stimulation and onset of pupillary constriction. Bold indicates signifcant diferences between patients with and without MLS

**Table 3:** Receiver-operating characteristics (ROC) analyses of pupillary parameters associated with development of midline shift (MLS) and prognostic value (i.e., sensitivity, specificity, positive predictive value, negative predictive value) of pupillary parameters for development of MLS ≥ 5 mm in 122 patients with endovascular treatment (EVT) due to large vessel occlusion (LVO) of the anterior circulation

	ROC analysis AUC (95% CI)	ROC analysis *p* value	ROC analysis calculated cut-off value	No midline shift	Midline shift ≥ 5 mm	Sensitivity % (95% CI)	Specificity % (95% CI)	Positive predictive value % (95% CI)	Negative predictive value % (95% CI)
Day 1 after EVT of anterior circulation LVO									
Per-change	0.606 (0.565–0.646)	< 0.0001	12.5%	472/1727 (27.3%)	104/223 (46.6%)	46.6 (40.0–53.4)	72.7 (70.5–74.8)	18.1 (15.0–21.5)	91.3 (89.7–92.7)
CV	0.584 (0.545–0.622)	< 0.0001	0.75 mm/s	768/1811 (42.4%)	129/233 (55.4%)	55.4 (48.7–61.8)	57.6 (55.3–59.9)	14.4 (12.2–16.9)	90.9 (89.1–92.5)
DV	0.634 (0.596–0.672)	< 0.0001	0.34 mm/s	746/1578 (47.3)	153/223 (68.6%)	68.6 (62.0–74.6)	52.7 (50.2–55.2)	17.0 (14.7–19.7)	92.2 (90.2–93.9)
Lat	0.514 (0.471–0.558)	0.512	0.19 s	1486/1543 (96.3%)	186/201 (92.5%)	92.5 (87.8–95.6)	3.7 (2.8–4.8)	11.1 (9.7–12.8)	79.2 (67.7–87.5)
Mean-delta per-change	0.757 (0.626–0.887)	0.003	0.46%	45/100 (45.0%)	11/13 (84.6%)	84.6 (53.7–97.3)	55.0 (44.8–64.9)	19.6 (10.7–32.8)	96.5 (86.8–99.4)
Mean-delta CV	0.828 (0.719–0.938)	< 0.0001	0.03 mm/s	41/104 (39.4%)	12/13 (92.3%)	92.3 (62.0–99.6)	60.6 (50.5–69.9)	22.6 (12.7–36.5)	98.4 (90.5–99.9)
Mean-delta DV	0.709 (0.558–0.860)	0.015	0.001 mm/s	26/96 (27.1%)	8/13 (61.5%)	61.5 (32.3–84.9)	72.9 (62.7–81.2)	23.5 (11.4–41.6)	93.3 (84.5–97.5)
Mean-delta Lat	0.459 (0.270–0.648)	0.643	-0.03 s	93/94 (89.9%)	11/12 (91.7%)	91.7 (59.8–99.6)	1.1 (0.1–6.6)	10.6 (5.7–18.5)	50.0 (2.7–97.3)
Day 2 after EVT of anterior circulation LVO									
Per-change	0.672 (0.611–0.733)	< 0.0001	20.5%	225/433 (52.0%)	59/74 (79.7%)	79.7 (68.5–87.8)	48.0 (43.3–52.9)	20.8 (16.3–26.1)	93.3 (88.9–96.1)
CV	0.695 (0.639–0.752)	< 0.0001	0.78 mm/s	135/470 (28.7%)	45/75 (60.0%)	60.0 (48.0–70.9)	71.3 (66.9–75.3)	25.0 (19.0–32.1)	91.8 (88.4–94.3)
DV	0.693 (0.636–0.749)	< 0.0001	0.43 mm/s	185/374 (49.5%)	61/74 (82.4%)	82.4 (71.5–90.0)	50.5 (45.4–55.7)	24.8 (19.6–30.8)	93.6 (89.0–96.4)
Lat	0.536 (0.452–0.619)	0.358	0.22 s	296/371 (79.8%)	45/65 (69.2%)	69.2 (56.4–79.8)	20.2 (16.3–24.7)	13.2 (9.9–17.4)	78.9 (69.1–86.4)
Day 3 after EVT of anterior circulation LVO									
Per-change	0.657 (0.588–0.725)	< 0.0001	19.5%	160/302 (53.0%)	52/63 (82.5%)	82.5 (70.5–90.6)	47.0 (41.3–52.8)	24.5 (19.0–31.0)	92.8 (87.2–96.2)
CV	0.709 (0.645–0.773)	< 0.0001	1.21 mm/s	179/326 (54.9%)	56/63 (88.9%)	88.9 (77.8–95.0)	45.1 (39.6–50.7)	23.8 (18.6–29.9)	95.5 (90.5–98.0)
DV	0.744 (0.678–0.809)	< 0.0001	0.39 mm/s	122/279 (43.7%)	50/55 (90.9%)	90.9 (79.3–96.6)	56.3 (50.2–62.1)	29.1 (22.5–36.6)	96.9 (92.6–98.9)
Lat	0.558 (0.454–0.662)	0.210	0.15 s	270/271 (99.6%)	38/46 (82.6%)	82.6 (68.0–91.7)	0.4 (0.0–2.4)	12.3 (9.0–16.7)	11.1 (0.6–49.3)
Days 1–3 after EVT of anterior circulation LVO									
Per-change	0.623 (0.593–0.654)	< 0.0001	17.5%	1190/2462 (48.3%)	238/360 (66.1%)	66.1 (60.9–70.9)	51.7 (49.7–53.7)	16.7 (14.8–18.7)	91.2 (89.6–92.7)
CV	0.618 (0.589–0.647)	< 0.0001	1.18 mm/s	1766/2607 (67.7%)	316/371 (85.2%)	85.2 (81.1–88.6)	32.3 (30.5–34.1)	15.2 (13.7–16.8)	93.9 (92.0–95.3)
DV	0.656 (0.628–0.685)	< 0.0001	0.35 mm/s	1022/2231 (45.8%)	250/352 (71.0%)	71.0 (65.9–75.6)	54.2 (52.1–56.3)	19.7 (17.5–22.0)	92.2 (90.6–93.6)
Lat	0.526 (0.490–0.563)	0.130	0.19 s	2107/2185 (96.4%)	281/312 (90.1%)	90.1 (86.1–93.0)	3.6 (2.8–4.5)	11.8 (10.5–13.1)	71.6 (62.0–79.6)

*ROC*, Receiver-operating characteristics analysis; *AUC*, area under the curve; *CI*, confidence interval; *EVT*, endovascular treatment, *LVO*, large vessel occlusion; *Per-change*, percentage change of aperture, constriction percentage, i.e., maximum size minus minimum size divided by maximum size; *CV*, constriction velocity, amount of constriction divided by the duration of the constriction; *DV*, dilation velocity, amount of pupil size recovery divided by the duration of the recovery; *Lat*, light reflex latency, time difference between initiation of retinal light stimulation and onset of pupillary constriction; mean delta, intra-individual differences between successive pairs of assessed pupillary parameters

Best discriminative thresholds, i.e., cut-off values indicating an imminent risk of developing BE, were then calculated using ROC-analyses for the entire monitoring period and for each day after ICU admission, respectively. AUCs for the different dynamic pupillary parameters increased with time, reaching maximum values on day 3 (except for per-change, which was highest on day 2) (Table 3). Best discriminative thresholds for each parameter and day of monitoring are listed in Table 3. ROC-analyses and AUC-values for mean-*Deltas* of dynamic parameters on day 1 of monitoring (except for Lat) also yielded significant associations with development of MLS [AUC (95% CI): day 1: mean-delta-per-change: 0.76 (0.63–0.89), *p* = 0.003; mean-delta-CV: 0.83 (0.72–0.94), *p* < 0.0001; mean-delta-DV: 0.71 (0.56–0.86), *p* = 0.015; mean-delta-Lat: 0.46 (0.27–0.65), *p* = 0.643, Table 3].

### Prognostic value of pupillary parameters for identification of patients with midline shift

Sensitivity for the different discriminative thresholds of dynamic pupillary parameters increased with time, reaching maximum values on day 3. Sensitivity of calculated cut-off values for each parameter and day of monitoring are listed in Table 3. Specificity for the different discriminative thresholds of dynamic pupillary parameters did not show a clear trend over time, and ranged between 45.1 and 72.7% (Table 3).

For calculated cut-off values of mean-*Deltas*, sensitivity (95% CI) and specificity (95% CI) on day 1 were: 84.6% (53.7–97.3) and 55.0% (44.8–64.9) for a threshold of < 0.46% for mean-delta-per-change [OR (95% CI) 6.72 (1.42–31.9), *p* = 0.017], 92.3% (62.0–99.6) and 60.6% (50.5–69.9) for a threshold of < 0.03 mm/s for mean-delta-CV [OR (95% CI) 18.44 (2.31–147.24), *p* < 0.001], and 61.5% (32.3–84.9) and 72.9% (62.7–81.2) for a threshold of < 0.001 mm/s for mean-delta-DV [OR (95% CI) 4.31 (1.29–14.37), *p* = 0.017], respectively (Table 3).

PPVs for all cut-off values ranged between a minimum of 11.1% on day 1 and a maximum of 29.1% on day 3, which suggests that all tested parameters are not suited as early predictors of BE and MLS. PPVs of cut-off values of mean-*Deltas* on day 1 were similarly low (10.6–23.5%), making them equally unsuited as predictive parameters for impending BE.

### Prognostic value of pupillary parameters for identification of patients without midline shift

In contrast to PPVs, NPVs were higher for all calculated cut-off values and climaxed on day 3 (with exception of per-change, which was highest on day 2). Calculated maximum NPVs were: 93.3 (88.9–96.1), 95.5 (90.5–98.0), and 96.9 (92.6–98.9) for per-change, CV, and DV, respectively (Table 3). NPVs for calculated cut-off values of mean-*Deltas* on day 1 after EVT were similarly high: (i) for mean-delta-per-change: 96.5% (86.8–99.4) for a threshold of < 0.46%, (ii) for mean-delta-CV: 98.4% (90.5–99.9) for a threshold of < 0.03 mm/s, (iii) for mean-delta-DV: 93.3% (84.5–97.5) for a threshold of < 0.001 mm/s—suggesting that these parameters adequately and early identify patients not at risk of developing BE. Again, Lat seems a less reliable parameter, as NPVs of mean-deltas on day 1 were considerably lower, i.e., 50.0% (2.7–97.3) for a threshold of > -0.03 s (Table 3).

## Discussion

To the best of our knowledge, this is the first study to introduce additional diagnostic and clinical significance of automated pupillometry in the very early phase after LVO-EVT, i.e., before routine imaging displays whether the patient develops malignant infarction or not.

We here demonstrate that (i) the dynamic parameters of the pupillary light reflex (CV, DV, and per-change) show a significant association with the development of MLS after EVT. On day 3 after EVT, i.e., when neuroimaging already revealed the actual size of infarction, but also already on day 1 (and 2) post-stroke, these variables were significantly lower in patients with MLS on CT-scans on day 3–5 than in patients without development of detrimental infarction. (ii) Although automated pupillometry does not seem suited to reliably predict impending risk of MLS, (iii) the device adequately identified patients who did not develop MLS days after EVT, with an NPV of up to 98.4% (for mean-delta CV). Furthermore, we (iv) identified cut-off values for pupillary parameters which had similarly been suggested useful in the monitoring of patients with other manifestations of acute brain injury [13, 18]. We (v) also introduced thresholds for intra-individual changes in light reflex parameters, which may be less susceptible to confounding variables and therefore appear specifically robust in indicating absent risk of MLS development early after EVT.

During the past few years, LVO treatment improved by implementing better EVT-devices and more precise neuroimaging algorithms, by increasing expertise, and by extending the time-window from symptom onset for eligible patients for EVT [21, 22]. Yet, there is still a notable number of patients who despite EVT develop LHI [23]. LHI patients at risk of developing BE should be identified as soon as possible to implement treatment options such as osmotic agents or decompressive hemicraniectomy [24]. Yet, monitoring of patients after EVT on neurological ICUs can be challenging. Neurological deterioration as first sign of increasing ICP and developing BE can barely be examined in analgosedated patients, and invasive ICP monitoring is not regularly recommended in ischemic stroke patients [1]. As the actual size of infarction remains uncertain until the follow-up imaging, the occurrence of LHI and developing BE is often diagnosed with a time delay that may result in irreparable brain damage and delayed implementation of rescue therapies.

Pupillary reactivity is known to be sensitive to changes in ICP [12, 16], and previous studies showed correlations of automated pupillary assessments in neurological ICU patients with ICP elevations [12, 18].

However, the majority of these studies focused on ICP elevation and the prognostic value of automated pupillometry as an add-on tool for patients with either intracranial bleeding, acute traumatic brain injury, or heterogeneous causes of acute brain injury, respectively [12, 14, 17]. Suggested cut-off values of pupillary parameters that may prompt toward impending herniation may not be applicable in patients with a different vascular disease and a different pathophysiological mechanism of BE development. Moreover, most research focused on the neurological pupillary index (NPi), which comprises a metric of all quantifiable pupillary parameters in one algorithm [12]. Yet, the light reflex results from the interaction of sympathetic and parasympathetic pathways which are represented by specific pupillometry parameters, and have been shown to be prone to external influences to a different extent. [25]. Although the NPi is a widely used parameter that has been demonstrated to be a useful metric in multiple studies, we here focus on dynamic pupillary parameters which may be less susceptible to confounding factors of intensive-care treatment, e.g., GA [18].

Similar to previous studies [14, 18], we found significant differences regarding all analyzed dynamic pupillary parameters but Lat between the two groups of patients with and without MLS. The differences were not only seen on each individual day after EVT, but also over the entire monitoring period of 3 days. These results are consistent with those of previous analyses of patients with acute brain injury [14, 17, 18], indicating once again that developing BE and rising ICP impairs normal pupillary function in a way that may be detected by automated pupillometry.

The thresholds for specific parameters derived from ROC-analyses here are also similar to thresholds calculated in the other studies [18]. Furthermore, in our study, we calculated thresholds for each individual day after EVT, making them on the one hand slightly less feasible, on the other hand more precise and more comparable among different patient groups, since they each reflect the comparatively homogenous patient population of one particular day. This aspect is underlined by the fact that those thresholds improved over time, reflecting the fact that already on the second day after EVT, a large proportion of patients were awake, conscious, spontaneously breathing, and analgosedation did not hamper pupillary function any more.

Another new aspect of our study is the introduction of delta-values as mean values of intra-individual deltas for each day of monitoring, assuming that changes between a pair of values better account for each patient’s individual risk of developing BE than comparing absolute values between different patients. This mathematical approach may further reduce the interference of confounders as these values should be robust to external influences. Group comparison of delta-values in patients with and without MLS also showed significant differences except for Lat. While this approach is so far not feasible—without automated pupillometers calculating and displaying delta-values at the moment—it bears the potential of an additional benefit of automated pupillometry in the future after validation in larger and prospective studies.

Yet, sensitivity and specificity analyses for best discriminative thresholds did not show convincing results regarding early prediction of impending BE after LVO-EVT. While sensitivity for all assessed dynamic pupillary parameters but Lat reached acceptable values even above 80% on the third day of monitoring, specificity was continuously low, questioning the clinical value of these parameters as early detection tool for patients with BE. Furthermore, the prognostic value of automatically assessed dynamic pupillary parameters seems unsatisfying as PPVs ranged from 11.1% to a maximum of 29.1% only. These results are in line with a previous study that demonstrated differences in pupillary reactivity between patients with and without ICP elevation without proving clinical value of automated pupillometry for prediction of ICP crises [18].

The additional clinical value of automated pupillary assessments in LVO patients after EVT seems to derive from the high NPVs. NPVs ranged from 90.9 to 96.9% in all dynamic parameters except Lat on every individual day as well as when measured throughout the whole period of 3 days. Using the calculated thresholds for each day could thus make pupillometry a useful tool for detection of patients who are unlikely to develop BE and who may not need repetitive CTs aside from the routine control-imaging procedure.

It is arguable that on day 2 and 3 after EVT, the patients’ conditions might be very heterogeneous concerning their state of consciousness and concomitant sedating medication with some patients still being on ventilator-support and some patients already awake, breathing spontaneously. This emphasizes the importance of our data from the first day after EVT. During the first 24 h after EVT, level of consciousness, duration of ventilation, and treatment with analgosedation and other medication were very alike among all patients, thus constituting a very homogeneous population. Already on the first day of measurement, NPVs yield high values (91.2–93.9%), which underlines the suitability of automated pupillometry in LVO patients in these critical first hours after EVT when analgosedation makes neurological assessments impossible and quick therapy decisions upon early recognition of deterioration could have an impact on the patients’ survival and outcome.

Despite these aspects, our study has several limitations. First, specific thresholds calculated in our study are so far not feasible for clinical practice, especially since delta-values as expression of intra-individual changes are introduced here for the very first time and not displayed by pupillometers. Thresholds need to be validated in large prospective trials before they may be implemented into clinical practice. Such trials should pre-specify time-points for serial CT-scans assessing the prevalence of BE to calculate reliable correlations. These studies should further document exact duration and dosage of analgosedative medication and other factors that could affect pupillary light reflex. It is well known that especially the sympathetically mediated part of the pupillary light reflex is sensitive to analgosedative medication [16, 18, 25]. By focusing on dynamic, mainly parasympathetically mediated parameters, we tried to minimize this bias. Second, inclusion of patients who regain consciousness and begin to breathe spontaneously early after EVT may limit applicability and generalisability of our results, as on day 2 and 3 after EVT patients’ conditions might already be very heterogeneous, with some patients still being on ventilator-support and some patients already awake, breathing spontaneously. When developing BE and MLS, patients are less likely to regain consciousness well enough to be deemed suitable for early extubation after EVT. The majority of conscious patients on days 2 and 3 are therefore part of the group without MLS by default. Consequently, NPVs from day 2 onwards might yield lower results upon removal of the subgroup of patients who were already conscious and spontaneously breathing, challenging validity of predictive values in this subgroup. Yet, this aspect emphasizes the importance of our data from the first day after EVT, as described above. Third, the retrospective character of our study limits reliability of calculated cutoffs, which stresses once more the need for a larger, prospective trial before certain thresholds can be put into practice. Fourth, it is worth mentioning that monitoring of the pupillary light reflex with automated pupillometers was initiated only after ICU admission, i.e., after EVT. However, implementation of automated pupillometry already in the emergency room may permit establishing “baseline” values in patients before EVT. Furthermore, since the degree of recanalization represented by TICI score (thrombolysis in cerebral infarction) has been shown to be associated with outcome [26], it has been suggested that the same applies for development of BE [19]. While the number of patients with a TICI score of 2a or less was significantly higher in our group with MLS compared to the group without MLS (30.8% versus 6.4%, *p* = 0.0155, Table 1), the number of patients with poor TICI scores was too small to perform analyses to evaluate significance of prospective pupillometry parameters in this specific subgroup. Yet, this question should be addressed in larger validation studies, since recanalization success may influence pretest probability for BE development. Finally, since we did not correlate data with neurological-outcome parameters, clinical significance of automated pupillometry findings in patients after LVO-EVT remains unclear.

In conclusion, our findings suggest that automated pupillometry may be helpful to early identify patients who are not at risk to develop BE after LVO-EVT. Prospective studies need to validate whether this bed-side technique harbors the potential to reduce follow-up CT-imaging.

## Data Availability

Data may be shared by writing to the corresponding author.
